# Differences in Subjective Well-Being between Formal and Informal Workers in Urban China

**DOI:** 10.3390/ijerph20010149

**Published:** 2022-12-22

**Authors:** Gengzhi Huang, Yanshan Yang, Yubing Lei, Jiangmin Yang

**Affiliations:** 1 School of Geography and Planning, Sun Yat-sen University, Guangzhou 510275, China; 2 Southern Marine Science and Engineering Guangdong Laboratory, Zhuhai 519082, China

**Keywords:** subjective well-being, happiness, formal employment, informal employment, urbanization, China

## Abstract

This paper examines the relationship between different types of employment and subjective well-being with a focus on informal employment. The China Labor-force Dynamics Survey (CLDS) for three selected years (2012, 2014 and 2016) shows an upward trend in the subjective well-being of urban workers in the 2010s. However, although the gap in subjective well-being between formal and informal workers narrowed, informal workers’ subjective well-being was still lower than their formal counterparts. Factors affecting the subjective well-being of formal and informal workers and their different effects were revealed to explain this difference. The subjective well-being of informal workers is significantly related to their informal status of employment, economic conditions (such as income and working hours), human capital, social capital (such as perceived social justice and perceived community connectedness) and urban environment. The paper enhances the understanding of people’s subjective well-being by differentiating informal/formal segments of working populations.

## 1. Introduction

Since the beginning of the 21st century, both developed and developing countries have undergone tremendous changes in economic conditions that reveal the basic material needs of their citizens. In this regard, both governments and individuals would like to achieve improved social development while maintaining economic growth [1,2]. In recent years, urbanization in China has made significant progress. In 2020, its urbanization rate reached 63.9%, which is higher than the world average [3,4]. On this basis, the Chinese government is now striving for higher-quality urbanization.

According to the World Values Survey, from 1990 to 2007, there was first a drop and then a slow increase in the subjective well-being of Chinese people [5]. Considering this result, Easterlin proposed that with an increasing unemployment rate and disappearing social welfare and income inequalities, the subjective well-being of Chinese people has been stagnating or decreasing during the period of economic transformation and development, and emphasized that employment and social welfare are important for improving subjective well-being in society [5]. However, while this argument offers some food for thought regarding how to improve overall subjective well-being in China, it also raises two questions. First, in China, a developing country, to what extent can employment and social welfare improve the quality of social development during continuous national economic growth? Second, for the population with low income, unstable employment, and lack of social welfare coverage, will their subjective well-being be much lower than that of the rest of society, and is there any other explanation for their relatively lower subjective well-being?

In order to answer the above questions, this research investigates the relationship between different types of employment and subjective well-being with a focus on informal employment. Data from social surveys on Chinese workers during the second decade of the 21st century will be used to analyze and compare the levels of subjective well-being among different types of workers to serve as the starting point for our analysis. Second, regarding the selection of determinants for subjective well-being, apart from informal employment, we also pay attention to the interaction between individual and environmental factors. Third, based on the comparison of the heterogeneity among workers, we aim to reveal the key factors of higher subjective well-being for informal workers to ensure the alignment between the development of the informal sector and ongoing high-quality urbanization in China. Fourth, we examine the possible interference of selection biases in informal employment and explore how to reduce workers’ unhappiness induced by informal employment in order to improve the policy environment.

## 2. Literature Review

Subjective well-being was first introduced by Diener, which measures people’s self-reported evaluation of their own life, including cognitive judgments and emotional responses [6]. A large amount of research mainly focused on the measurements and determinants of subjective well-being. A self-report rating scale is a commonly used method to measure subjective well-being [7]. The existing literature has demonstrated that subjective well-being is determined by both internal and external factors [8]. Early studies focused on the relationship between subjective well-being and demographic characteristics such as age, gender, health, marriage, and religion [9,10,11,12,13,14,15]. In recent studies, more attention has been paid to how external factors (economic and non-economic factors) affect subjective well-being. Community and societal factors are associated with subjective well-being, including perceived social justice and perceived community security and community connectedness, which reflect social capital [16,17]. In addition, the urban environment has also been shown to influence subjective well-being. Better air quality, more green coverage and a well-maintained transport system can improve an individual’s well-being by fostering social interaction and safety [18,19].

Later on, as the meaning of a “good life” in subjective well-being came to be widely applied in evaluations of social development [11], there were more heated discussions on the relationship between changes in economic factors and subjective well-being. Income and unemployment are the most commonly discussed factors [20,21]. Veenhoven believes that happiness is closely linked to economic conditions and that there is a difference in happiness among countries with different income levels [2,22]. Vanlente and Berry’s study of Latin America and the United States confirmed that personal income and per capita national income has a positive effect on subjective well-being while being unemployed is negatively related to an individual’s happiness [23]. However, there are limited studies on the effect of the type of employment, especially in developing countries. With rapid changes in the global economic environment, informal employment is continuously expanding in developing countries [24,25]. According to a recent report from the International Labor Organization (ILO), two billion or 61.2% of the global employed population are informal workers [26]. The proportion of informal employment varies in different regions. While the proportion of informal employment in developing countries reaches 69.6%, it is 18.3% in developed countries [26]. Since the adoption of reform and opening-up policies in 1978, China has seen the informalization of employment and a remarkable rise in informal employment. It is estimated that 183 million workers were engaged in the informal economy in 2018, which accounts for about 9% of the two billion informal workers worldwide [27]. However, the relationship between informal employment and subjective well-being is unclear. On the one hand, some scholars argue that as informal employment signifies low-quality work, evidently unstable income, higher employment risks, and lower labor and social security [28,29], such expansion could lead to lower levels of subjective well-being [30,31,32,33]. On the other hand, based on case studies of informal employment in Mexico, India, Bosnia and Herzegovina, a number of scholars have pointed out that informal employment is a choice made by workers in developing countries to pursue a better quality of life in the face of low-quality conditions of national development [34]. When more people opt for the informal sector, they are, in fact, seeking higher earnings, more freedom and autonomy [35,36].

In addition, studies suggest the mediating and moderating effect of other factors need to be taken into consideration when examining the impact of informal employment on workers’ happiness. Social inequality [32], income [37] and unemployment probability [38] play a mediating role in the relationship between informal employment and subjective well-being, while other factors, such as the labor system, can moderate some mediating effect [30]. The study by Liu et al. shows that lower labor income reduces the informal workers’ happiness, while social security moderates this negative impact. As a result, an inverted U-shaped relationship is observed between flexible employment and residents’ subjective well-being [37]. Chen et al. examined the heterogeneity of the relationship between informal employment and subjective well-being and found that informal employment causes a lower level of happiness under the mediating effect of unemployment probability and social insurance level [38]. However, although scholars have paid attention to informal work, the difference in subjective well-being between formal and informal workers is rarely studied. There is a disparity between formal and informal employment in terms of employment status, economic income and social security [39]. This disparity might lead to the difference in subjective well-being between these two groups of workers. This paper thus takes up the attempt to examine the subjective well-being of workers by highlighting the difference between formal and informal employment. As an increasing number of workers in urban areas are engaged in informal employment due to the flexibilization of labor markets in currently neoliberalizing economies, it is important to differentiate between formal and informal workers. Moreover, informal employment has been registered as a major challenge to inclusive economic growth and decent work by the UN in the 2030 Sustainable Development Goals [40]. This paper is helpful for understanding the social challenge and inequality resulting from the informalization of employment sectors.

In order to examine the question above, it is necessary to define informal employment. Currently, there are still controversies surrounding the definition of informal employment. On the one hand, this is because informal employment is not captured by official statistical data, while on the other hand, the term carries distinctively different meanings in different contexts. In the dualist view, informal employment refers to short-term economic behaviors adopted to increase income [41,42] and will eventually be absorbed by the formal economy and disappear [43]. However, from a neo-Marxist perspective, informal employment is a form of employment to circumvent regulation and reduce administrative costs in the formal capitalist economy [44]. Neoliberalists believe that such evasion of regulation is an indication of workers’ initiatives to realize self-value [45]. Although these definitions have not been adopted in demographics on informal employment, the distinction between them can help us identify various types of informal workers under the system of the formal urban economy, such as porters and construction workers, which should be highlighted in defining informal employment.

Data limitation presents a challenge to the study of informal employment as it is not covered by the state’s regular statistics. In promoting quantitative research on informal employment, the International Labor Organization (ILO) proposes four dimensions for defining informal workers: the nature of the corporation, the size of the enterprise, accounting, and whether labor contracts are signed with employees [46]. In view of the complexity of the informal sector in developing countries, the ILO also specifies that each country and region can categorize informal employment according to the characteristics of the position and entity [47]. In China, based on international standards and the conditions of informal employment in the country, many scholars have argued that employees in the informal sector should include self-employed persons, employees in private enterprises without labor contracts, and a great number of informal workers in unregistered enterprises [48,49]. Wu and Cai consider employees of temporary work, employed workers without formal labor contracts, self-employed workers, and home helpers as informal workers and establish three statistical criteria to satisfy the different research requirements [50]. In light of the fact that informal employment is not covered in the formal institution, informal workers should be characterized by “no formal labor contracts” and “no participation in social security” [51]. In our research, with the bundled social security system in China, participation in social security mainly refers to whether a worker has enrolled in the urban employee basic medical insurance or the basic endowment insurance for urban workers.

## 3. Research Design

### 3.1. Regression Model

The ordered logistic model, which is also called the cumulative logistic model, is often employed in situations where the dependent variable is ordered and discrete, while the independent variable can be continuous, discrete, categorical, or ordinal [52]. In this research, subjective well-being, the dependent variable, is measured in integers from 1 to 5 and is, therefore, an ordered discrete variable, which meets the requirement of this type of regression [53].

According to the mathematical principle of the model [54], ordered logistic regression uses a number of binary logistic regressions to describe the odds of each category against the reference. The formula for the transformation between models is as follows:

If there are *n* categories in the dependent variable and they take place following the order of *Y* = 1, 2,…, *n*, suppose the conditional probability *p* = *P*(*y* = 1|*x*_1_, *x*_2_,……, *x_w_*) is the probability of the observed category as compared to an event *x* occurring, then the cumulative probability of *n* or fewer categories occurring can be calculated using:

*P*(*Y* ≤ *n*) = *P*(*y* = 1|*x*_1_) + *P*(*y* = 1|*x*_2_) +……+ *P*(*y* = 1|*x*_w_) (1)

Denote the *w* independent variables in the model as *X* = (*x*_1_, *x*_2_, ……, *x*_w_), and the cumulative logit(*Y* ≤ *n*) function becomes:

(2) $$\mathrm{logit}\;[P(Y\;\leq \;n)]=\ln\;\frac{P(Y\leq n|x)}{1-P(Y\leq n|x)}\;=\ln\;\frac{P(y=1|x_{1})+P(y=1|x_{2})+\ldots \ldots +P(y=1|x_{w})}{P(y=1|x_{w+1})+P(y=1|x_{w+2})+\ldots \ldots +P(y=1|x_{w})}$$

According to Formula (2), the cumulative logit(*Y* ≤ *n*) function is the log ratio of the two cumulative probabilities and can represent the probability that the dependent variable *Y* equals to or is less than *n* categories. Furthermore, as there are *n* ordered categories, there can, in fact, be at most *n* − 1 logit functions in an ordered logistic regression. Thus, the linear expression of the ordered logistic functions is as follows:

(3) $$\mathrm{logit}\;[P(Y\;\leq \;n)]=\left\{\begin{array}{c} \beta_{1,0}+\beta_{1,1}x_{1}+\ldots \ldots +\beta_{1,w}x_{w}\\ \ldots \ldots \\ \beta_{n-1,0}+\beta_{n-1,1}x_{1}+\ldots \ldots +\beta_{n-1,w}x_{w} \end{array}\right.$$

The transformation between the probability of the model and regression functions can be achieved with the following equation:

(4) $$P(Y\;\leq \;n)=\frac{exp\left(\beta_{w,0}+\beta_{w,1}X_{1}+\ldots \ldots +\beta_{w,n}X_{w}\right)}{1-exp\left(\beta_{w,0}+\beta_{w,1}X_{1}+\ldots \ldots +\beta_{w,n}X_{w}\right)}$$

### 3.2. Selection Bias in Informal Employment and Relevant Tests

Existing studies point out that whether workers are from the formal or informal sector is not randomly distributed but is influenced by other factors, including gender, marital status, and education [55]. This implies a biased result in the investigation of the causal relations between informal employment and subjective well-being. Propensity Score Matching is a technique to study relations between core variables after unbiased estimation of control variables in the model and requires that the matched control variables pass a balance check [56]. For more robust testing of causality between informal employment and subjective well-being, we adopted nearest neighbor matching, radius matching, and core matching to ensure the reliability of the result. We also examined the Average Treatment Effect (ATT) on treated individuals and the significance level of the treatment group.

### 3.3. Data Sources

Our research involves surveys and statistical data. The survey data come from the China Labor-force Dynamics Survey (CLDS) conducted by the Center for Social Science Survey at Sun Yat-sen University. The center used a multi-stage, stratified probability sampling proportionate to the labor size to conduct the survey from July to September 2012, 2014, and 2016 in 29 provinces and municipalities (Hong Kong, Macao, Taiwan, Tibet, and Hainan province were excluded). The data were collected from village committees (or residents’ committees), families, and family members aged between 15 and 64 (eligible for work). In terms of the sample size, there were 16,253, 23,594, and 21,086 cases for the surveys in 2012, 2014, and 2016, respectively. There are no official statistics on informal employment in China. The CLDS provides the latest sufficient individual-level labor data that covers the information on both workers’ employment status and their subjective well-being. Furthermore, because the release of official statistics from the Chinese government tends to be delayed by one year, we obtained the 2016 data from the 2017 *China Statistical Yearbook*. The statistical data in our research also stem from the 2017 *China Statistical Yearbook* and local statistical yearbooks from the year 2017 for the prefecture-level cities. We obtained the prices for newly built houses in prefecture-level cities from June to September 2016 from the Xitai real estate data (https://www.creprice.cn (accessed on 8 June 2021)).

While we strived to minimize the sample loss, the two selected sample groups are inherently different. First, we selected the urban working population with non-agricultural household registration and between the age of 16–64 (the minimum legal age to work in China is 16) in the three-year survey data and deleted cases with missing values and outliers. From this process, we obtained 3220, 5258, and 4440 valid samples for each year, respectively, to study the change in subjective well-being among workers in urban China. Second, for regression modeling, we selected the 2016 data on workers from CLDS. Notably, it is required to retain as many samples as possible and to ensure the genuineness and reliability of the data. Therefore, after removing missing values and outliers, we deleted cases that did not fit with reality in terms of economic factors, including income and weekly working hours. Ultimately, there were 3855 cases. The processed data still covered 29 provinces and municipalities, and the mean values of relevant variables were more or less in line with the actual situation, which conforms to the scientific nature of statistical inference.

### 3.4. Variables Specification

#### 3.4.1. Informal Employment

Informal employment is not only the key independent variable for constructing the baseline regression model but also a crucial criterion for categorizing the subjective well-being of different types of workers. According to the definition of informal workers in the Chinese context, we have three types in total: (1) private business owners and self-employed persons who have no legal person status, no employees, or hire less than five people (in accordance with the international standards) and do not sign contracts with employees; (2) employees without labor contracts and those who have signed labor contracts but are not covered by the basic endowment insurance for urban workers or the urban employee basic medical insurance; and (3) self-employed persons with no fixed work contents. Altogether, we had 2312 cases of informal workers in 2016, accounting for about 52% of the total sampled employees. This proportion is basically in line with the size of the informal employment as measured in existing studies. Based on the categories in informal employment, we grouped them into employed workers (employees) and self-employed workers (private business owners, individually-owned business owners, and self-employed persons), so that there are 1714 employed and 598 self-employed informal workers.

#### 3.4.2. Subjective Well-Being

Subjective well-being is the dependent variable in this research, measured by asking respondents, “in general, are you happy with your life at the moment?” to indicate the subjective feelings of urban workers with respect to their current life situation. We adopted a five-point scale ranging from 1 = “very unhappy” to 5 = “very happy”. However, the scale used in the 2012 survey was a six-point scale from 1 to 6, which does not correspond to those in the 2014 and 2016 surveys. Therefore, to employ a common scale, we normalized the values of subjective well-being measured in 2012 to project them onto a 5-point scale.

#### 3.4.3. Control Variables

As shown in Table 1, in the selection of control variables that may affect workers’ subjective well-being, we referred to existing literature and found that several demographic variables can affect individuals’ happiness, such as age, health condition, gender, religion and marital status. Concerning the impact of age, the subjective well-being of a few middle-aged workers does not appear to be relatively higher, as we expected. In this regard, we introduced the squared value of age in our analysis. In order to match the original data structure, the age variable was transformed into age squared/100. Furthermore, in cities, although the influence of the Chinese hukou system on workers’ livelihoods has weakened to a great extent, migration between regions might affect individuals’ lives. The hukou system in China is a household registration system that defines the local residents who are entitled to get access to social welfare provided by the local government. Migrants usually have no hukou in the destination city and are, therefore, unable to get access to local social welfare systems. Thus, we considered the factor of migration.

For urban workers as our research subjects, economic conditions and work treatment can have a huge impact on happiness, so we considered annual income after accounting for housing prices, weekly working hours, education level, skill level, and perceived value in work. Specifically, annual income after accounting for housing prices is a measure using the difference in housing prices across regions to indicate levels of consumption. This is obtained by dividing annual income by urban housing prices to show the real disposable income of workers after eliminating the difference in consumption levels. In the meantime, working hours are of equal importance, and from common experience, long working hours will damage the subjective well-being of workers. Further, the accumulation of human capital endows workers with greater advantages at work, including higher levels of education, certain amounts of skills, social networks, and so on. A sense of value in work also refers to the self-value derived from the job in addition to economic income, such as gaining respect from others, expanding networks, and maximizing the application of one’s abilities.

Although social capital and urban environment variables may not play a significant role in determining subjective well-being as do other demographic and economic factors, justice in the social environment, closeness among residents in the community, safety in the residential area, adequate urban public infrastructure, and green coverage all have certain impacts on the effect of workers’ personal or economic factors on subjective well-being. In our research, the number of primary schools per 10,000 people, which is a key concern for workers, was chosen to indicate the soundness of urban public infrastructure allocation, while we employed green coverage of built-up areas to measure the greenness of cities. Furthermore, compared with the built-up areas within cities, the city size is a more macro-level indicator. According to the existing Standards for Categorizing City Sizes [57] and considering the requirements in our research, we divided cities into three tiers, ranging from small-and-medium-sized cities (with less than 1 million permanent residents), large cities (with between 1 and 5 million permanent residents), and mega-cities (with over 5 million permanent residents).

## 4. Results

### 4.1. Changes and Differences in Subjective Well-Being of Formal and Informal Workers

As shown in Table 2, in the sampled three years, the subjective well-being of urban workers in China was above average (3 scores) and close to the level of “relatively happy” (4 scores). Furthermore, as time passed, the happiness score increased, with subjective well-being rated 3.873 ± 0.874 in 2016. This indicates that since the beginning of the 21st century, Chinese urban workers have seen a significant improvement in their quality of life and subjective well-being. This result puts into question the unreliable prediction in earlier studies that the social welfare of workers in China’s economy would disappear or plateau, thus leading to a further drop in people’s happiness.

Nevertheless, it is undeniable that unequal development exists in the course of urbanization, which will further exacerbate the divergence in happiness among workers. In our research, the subjective well-being of informal workers was lower to a small extent than that of formal workers, while among informal workers, employees’ scores were higher than those of the self-employed. Additionally, among the four types of employment, formal workers had the highest level of subjective well-being, while self-employed informal workers had the lowest level. This shows that employment relationships covered by formal institutions are correlated with high levels of subjective well-being.

### 4.2. Average Effect Analysis on Workers’ Subjective Well-Being

Table 3 focuses on the influence of informal work on workers’ subjective well-being. By building continuous models based on variables of different dimensions, the model aims to investigate how economic factors and human capital influence subjective well-being after controlling for demographic factors. It also examines how social capital and the urban environment reshape that influence. Overall, by considering the demographics, economic factors, human capital, social capital, and urban environment, Model 3 in Table 3 has the largest pseudo R^2^, at 0.078, which means this model has the best fit.

As the results indicate, informal employment exerted a negative effect on workers’ subjective well-being, which is significant at the 5% level in Model 3. This shows that after controlling for demographic, economic, and environmental factors, informal work embodies employment inequality compared with formal employment. Furthermore, self-reported health conditions, marital status, perceived value in work, and social justice have a considerably greater influence on subjective well-being than other variables and are always significant at the 1% level. As such, these factors should be emphasized in the process of advancing better urban life. In terms of demographics, older workers tend to be less happy. Existing studies show that happiness is strongly U-shaped in age, which means middle-aged people tend to have lower levels of subjective well-being, and the turning point of happiness is around ages 35 to 53, with a bottoming out at an estimated 40 years old [58]. In general, female workers have a higher level of happiness than their male counterparts. Additionally, religion can, to some extent, increase subjective well-being. About 80% of the study sample are married, and they report higher levels of subjective well-being than the unmarried. This indicates that getting married can improve happiness. Despite a certain negative influence from migration, its effect on subjective well-being becomes insignificant after adding factors related to social capital and urban environment. Moreover, it has been proved that more human capital can improve the work environment. For instance, a higher level of education or certain skill sets will help workers fare better at work.

The link between income and subjective well-being has always been at the center of discussion. In our study, the higher the personal income, the happier the workers feel. Nevertheless, with the inclusion of other control variables, this positive effect turns from significant at the 1% level to insignificant, implying that the influence of income might be affected by other environmental factors. One possible explanation is that as the economic growth in China progresses to a certain level, workers may crave a better sense of identity, more welfare and security, and a more just social environment, in addition to receiving a corresponding amount of payments. Such craving reveals the change in individual needs in the post-materialist era and the search for a happy life in cities rather than suggesting an insatiable demand, as described by some scholars [59].

Geographic factors do not play a direct role in determining an individual’s subjective well-being, but their effects should not be neglected. In particular, against the background of promoting better urbanization, the geographical environment should be highlighted to improve individuals’ quality of life. The results show that more green coverage and a safer and more connected community environment can hugely add to the subjective well-being of workers, and the effects are significant at the 5% level. However, as an indicator of urban infrastructure, the number of primary schools per 10,000 population does not have a statistically significant relationship with workers’ happiness. This might be because although education is of great interest to workers, the current resource allocation in cities is not equalized, with little consideration of the different needs of people residing, living, and working in different areas of the cities. Therefore, in the future, policymakers should pay more attention to micro-level regional planning and search for a path to equalize basic public services in China. In the meantime, city size does not significantly affect workers’ subjective well-being. This might be because this variable has a broad spectrum of meanings compared to those indicating the internal urban environment so its effect on workers is relatively uncertain.

### 4.3. Different Analysis of Workers’ Subjective Well-Being in Formal and Informal Employment

The descriptive statistics show that the subjective well-being of informal workers is lower than both that of formal workers and the average of all workers. The above analysis also shows that there is still a significant negative causal relationship between informal employment and subjective well-being, even when the influential control variables are accounted for. This conclusion begs the question: what factors have led to the low subjective well-being of informal workers? Will these factors have an inverse effect on formal workers? To this end, we divided workers into formal and informal and included relevant variables that influence subjective well-being to examine the difference in effects between the two groups. As there is heterogeneity among employed and self-employed workers in the informal sector, we also considered the difference in effects between the two sub-categories to ameliorate the research on workers’ subjective well-being.

According to Table 4, regardless of the type of worker, health, marriage, self-value in work, and social justice are the topics of interest, which stresses the importance of these factors in affecting workers’ happiness. Meanwhile, by comparing the determinants across formal and informal workers, we found that apart from the above variables, education and community connectedness are crucial for the happiness of both formal and informal workers. However, education is not significant for the subjective well-being of self-employed informal workers. This might be because this population has relatively low education in general, and when it comes to technical work, those with certain skills will have more advantages than those with higher education.

Apart from the variables with the same influences on formal and informal workers, there also exist differences in effects. For the formal working population, age, gender, and perceived community safety are significant factors, while for informal workers, their effects are not significant. This can be explained by the fact that some informal workers have accumulated work experience as they age and that this increase in age can be regarded as an accumulation of human capital, which reduces the unhappiness of older informal workers. Although male informal workers may face a higher workload and more family pressure than females, they are able to meet their demand for work through various channels in a relatively flexible work environment. In this case, there is little difference in subjective well-being between men and women in informal employment. Furthermore, social capital positively and significantly affects workers’ subjective well-being, but informal workers may have less demand for community safety because informal employment tends to occur in urban villages or communities in old towns with high safety risks, large population flows, and complex social environments. On the other hand, informal workers seem to pay more attention to working hours, skill levels, and green coverage in cities. In contrast, the influence of these factors is relatively small for formal workers. This is because, without social security, long working hours can make informal workers feel more tired. Informal work is more similar to selling physical strength for money, and since the wages are not proportionate to their efforts, informal workers have lower levels of happiness. Finally, increased skill level can make up for the poor work treatment caused by the commonly low education among informal workers, while more green coverage tends to offer them a greater sense of happiness.

### 4.4. Robustness Testing

As mentioned above, there is likely to be self-selection in sampling informal employment, which results in bias when detecting the causal relations between informal employment and subjective well-being. Therefore, we employed propensity score matching to balance the differences in covariates between the treatment group (informal employment) and reference group (formal employment). The result is that there is still a negative causality, as indicated in the regression model, which suggests the regression analysis is reliable.

We realized that a distinct feature of the informal sector is the lack of coverage by formal institutions. As a result, workers in this sector must bear higher risks in work while receiving relatively little social security. In this sense, we introduced two factors on the social welfare environment closely related to workers’ interests, that is, the urban household registration system and the social security system, which can serve to test the finding. Due to the strong collinearity between informal employment and social welfare, we adopted an interaction term to cope with the relations between informal employment and social welfare. As indicated in Table 5, when informal workers settle their households in a city or enroll in the social welfare system in China, they can access better quality public services bundled with the systems of urban household registration and social security. Examples include sufficient education resources, medical and health services, and convenient public facilities, which will considerably mitigate the negative effect of informal employment. In the meantime, the result demonstrates that an improved institutional environment can eliminate the workers’ perceived injustice induced by their informal status of employment.

## 5. Discussion

Our study shows that social development in China has witnessed a delightful new era of progress compared with the early period of economic transformation. This trend is in line with the existing literature on subjective well-being during the current period in China [60]. In our study, perceived health condition, marital status, the perceived value of work and perceived social justice were shown to have rather significant impacts on workers’ subjective well-being. Workers with a higher level of subjective well-being are more likely to be healthy, married and consider their work valuable and the society equitable. Moreover, the positive effect of material pursuits (income) is decreasing, while spiritual pursuits (perceived value of work) are closely linked to higher subjective well-being [59,61]. This shows the significance of the non-materialistic pursuit of improving workers’ happiness. In addition, geographical factors are equally important in causing further changes in subjective well-being [62]. As sustainable development is increasingly valued in the construction and management of Chinese cities, improving green coverage and augmenting community safety and connectedness among residents can effectively increase urban workers’ happiness. Meanwhile, the role of urban public service infrastructure also reveals the pitfalls in resource allocation according to the number of permanent urban residents. Thus, it is advisable to pay attention to the heterogeneity of regions in urban infrastructure planning.

In view of employment, similar to previous studies, we found that informal employment negatively affects subjective well-being [63]. However, we believe that the expansion of such employment would not reduce the societal level of subjective well-being in China because social welfare can offset the perceived injustice among workers caused by informal employment. As social security coverage expands in China, the difference in employment might no longer become an obstacle for workers to gain more happiness. Nevertheless, considering the different levels of progress in implementing the social security system in cities, more empirical evidence is required to support this argument. However, we can still confirm that increased human capital will result in better treatment at work for both formal and informal workers. A higher level of education or skillset will be a considerable advantage in both formal and informal employment.

## 6. Conclusions

Our study examined the relationship between the development of informal employment and the overall societal level of subjective well-being in China. Based on the three-year social survey data in China within the second decade of the 21st century, we found that the current subjective well-being in Chinese society keeps increasing. In addition, the results show that informal employment negatively affects an individual’s subjective well-being, although this effect can be mitigated by the accumulated human capital, social capital, improved urban environment and the social welfare system. This study adds knowledge to the research on the subjective well-being of workers by differentiating formal and informal working populations in the context of the informalization of urban labor markets. We reveal the heterogeneity among workers’ subjective well-being by differentiating the type of employment and find that the internal and external determinants have different impacts on formal and informal workers. Specifically, informal workers’ subjective well-being is related not only to their informal status of employment but also to their economic conditions (such as income and working hours), human capital, social capital (such as perceived social justice and community connectedness) and urban environment.

This study has some policy implications. First, the government should focus on improvements in working conditions and the urban social environment since the importance of non-economic factors is increasing. It is necessary to create a high-quality and decent working environment and improve social justice and community connectedness. Second, there are determinants with different impacts on formal, informal employed and informal self-employed workers. Policies should be differentiated according to the types of workers affected. For instance, for formal workers and employed informal workers, the policies should focus on improving community security since it is a crucial element for their happiness. For workers who are doing easy-access self-employed jobs, the government should encourage them to receive further education or technical training, help them to improve their skills, and to achieve higher quality social development.

The study has some questions and limitations that need to be addressed in the future. We conducted our research using quantitative survey data and examined the workers’ happiness with econometric models, which are limited in explaining how people’s subjective well-being is formed. Thus, future efforts should be made to further investigate the subjective well-being of different types of workers and how the influencing factors work by using qualitative methods. Moreover, we measured subjective well-being by asking about respondents’ general life satisfaction. However, subjective well-being is a complicated system. Other dimensions, such as job satisfaction, should be taken into account in future studies.

## Figures and Tables

**Table 1:** Mean value of control variables for the 2016 sample.

Variables	Definition	Total N = 3855	Informal Workers N = 2312	Formal Workers N = 1543
Age	Value of age	40.57	40.89	40.10
Age squared/100	Value of age squared/100	17.57	17.84	17.18
Self-reported health conditions	Extremely unhealthy = 1; Unhealthy = 2; A bit healthy = 3; Healthy = 4; Very healthy = 5	3.91	3.92	3.90
Gender	Female = 0; Male = 1	0.55	0.55	0.55
Religion	No religious belief = 0; Have religious beliefs = 1	0.10	0.10	0.09
Marital status	Unmarried = 0; Married = 1	0.82	0.83	0.81
Migrant	Local resident = 0; Migrant = 1	0.23	0.25	0.20
Income after accounting for housing prices	Annual income/housing price	7.04	7.10	6.97
Working hours per week	Working hours in a week (hours)	44.95	47.89	40.54
Education level	Never went to school = 0; Primary school = 6; Junior high school = 9; Secondary specialized school or above = 12; Junior college or above = 16; Master = 19; Doctor = 22	12.26	11.81	12.95
Skill level	Have no certifications = 0; Have certifications = 1	0.34	0.29	0.42
Perceived value in work	Valueless = 1; Low value = 2; Valuable = 3; Relatively valuable = 4; Very valuable = 5	3.63	3.62	3.64
Perceived social justice	Extremely unfair = 1; Unfair = 2; Neutral = 3; Fair = 4; Extremely fair = 5	3.27	3.28	3.26
Perceived community connectedness	No connection = 1; Rarely connected = 2; Sometimes connected = 3; Often connected = 4; Closely connected = 5	3.26	3.28	3.22
Perceived community security	Extremely unsafe = 1; Unsafe = 2; A bit unsafe = 3; Safe = 4; Very safe = 5	3.02	3.03	3.01
Number of primary schools per 10,000 population	Number of primary school/10 thousand people	1.03	1.05	1.01
Green coverage of built-up areas	Green coverage area/urban built-up area (%)	40.81	40.58	41.16
City tier	Defined by the number of permanent urban populations, less than 1 million = 1; between 1 million and 5 million = 2; more than 5 million = 3	2.30	2.24	2.37

Source: Labor data is from the China Labor-force Dynamics Survey (CLDS) 2016 provided by the Center for Social Science Survey, Sun Yat-sen University. The city data is from the *China Statistical Yearbook* 2017, published by the State Statistics Bureau of China, and the housing prices are from Xitai real estate data (https://www.creprice.cn (accessed on 8 June 2021)).

**Table 2:** Comparison of workers’ subjective well-being in 2012, 2014, and 2016.

Year	Total (M ± SD)	Formal Workers (M ± SD)	Informal Workers (M ± SD)	Informal Workers (Employed) (M ± SD)	Informal Workers (Self-Employed) (M ± SD)
2012	3.664 ± 0.984	3.765 ± 0.935	3.594 ± 1.012	3.608 ± 1.002	3.534 ± 1.051
2014	3.823 ± 0.878	3.857 ± 0.887	3.811 ± 0.875	3.836 ± 0.864	3.733 ± 0.903
2016	3.873 ± 0.874	3.922 ± 0.869	3.841 ± 0.876	3.876 ± 0.845	3.741 ± 0.954

**Table 3:** Ordered logistic regression on subjective well-being based on sample data.

	Variables	Model 1	Model 2	Model 3
Key variables	Informal employment	−0.166 ***	−0.119 *	−0.132 **
Individual demographics	Age	−0.062 ***	−0.061 ***	−0.054 **
	Age squared/100	0.071 ***	0.076 ***	0.065 **
	Self-reported health conditions	0.563 ***	0.518 ***	0.436 ***
	Gender	−0.221 ***	−0.211 ***	−0.204 ***
	Religion	0.161	0.206 **	0.182 *
	Marital status	0.696 ***	0.756 ***	0.784 ***
	Migrant	−0.253 ***	−0.141 *	−0.085
Economic factors	Salary after accounting for housing prices	0.019 ***	0.010 **	0.005
	Working hours per week	−0.006 ***	−0.003 *	−0.003 *
Human capital factors	Education level		0.049 ***	0.051 ***
	Perceived value in work		0.438 ***	0.362 ***
	Skill level		0.147 ***	0.187 ***
Social capital factors	Perceived social justice			0.416 ***
	Perceived community connectedness			0.132 ***
	Perceived community security			0.213 ***
Urban environment factors	Number of primary schools per 10,000 population			0.043
	Green coverage of built-up areas			0.013 **
	City tier			−0.042
	Pseudo R^2^	0.035	0.057	0.078
	N	3855	3855	3855

Note: *, **, and *** indicate the confidence level at 10%, 5%, and 1%, respectively.

**Table 4:** Comparison across different employment types of effects on workers’ subjective well-being.

	Variables	Formal Employment	Informal Employment	Informal Employment (Employed)	Informal Employment (Self-Employed)
Individual demographics	Age	−0.084 **	−0.036	−0.026	−0.069
	Age squared/100	0.099 **	0.046	0.033	0.089
	Self-reported health conditions	0.400 ***	0.462 ***	0.450 ***	0.472 ***
	Gender	−0.304 ***	−0.134 *	−0.137	−0.232
	Religion	0.260	0.173	0.206	0.120
	Marital status	0.804 ***	0.797 ***	0.819 ***	0.849 ***
	Migrant	−0.058	−0.107	−0.152	0.009
Economic factors	Salary after accounting for housing prices	−0.003	0.012 *	0.017 *	0.006
	Working hours per week	0.000	−0.004 **	0.000	−0.008 ***
Human capital factors	Education level	0.050 ***	0.049 ***	0.066 ***	−0.007
	Perceived value in work	0.387 ***	0.339 ***	0.339 ***	0.416 ***
	Skill level	0.111	0.248 ***	0.102	0.734 ***
Social capital factors	Perceived social justice	0.373 ***	0.443 ***	0.405 ***	0.539 ***
	Perceived community connectedness	0.119 **	0.140 ***	0.140 ***	0.163 **
	Perceived community security	0.375 ***	0.099	0.047	0.234 *
Urban environment factors	Number of primary schools per 10,000 population	0.014	0.050	0.074	−0.040
	Green coverage of built-up areas	0.002	0.021 **	0.011	0.046 ***
	City tier	−0.174 *	0.030	0.106	−0.131
	Pseudo R^2^	0.078	0.080	0.078	0.103
	N	1543	2312	1714	598

Note: *, **, and *** indicate the confidence level at 10%, 5%, and 1%, respectively.

**Table 5:** Effects of informal employment and interaction between informal employment and social welfare on subjective well-being.

Regression or Matching Technique	Informal Employment ATT(SE)	Informal Employment * Urban Household Registration System ATT(SE)	Informal Employment * Social Security ATT(SE)
Logistic regression	−0.111 * (0.063)	−0.039 (0.067)	0.052 (0.132)
Nearest neighbor matching	−0.088 * (0.051)	−0.055 (0.041)	0.063 (0.097)
Radius matching	−0.071 ** (0.030)	−0.041 (0.030)	0.031 (0.058)
Core matching	−0.066 ** (0.029)	−0.042 (0.030)	0.053 (0.059)

Note: * and ** indicate the confidence level at 10% and 5%, respectively.

## Data Availability

Data used in this paper is from the China Labor-force Dynamics Survey (CLDS) by the Center for Social Science Survey at Sun Yat-sen University in Guangzhou, China. Please refer to http://css.sysu.edu.cn (accessed on 9 March 2020) or contact cssdata@mail.sysu.edu.cn for more information about the CLDS data.
